# Implantable cardioverter defibrillator implantation and degree of left ventricular scarring predict survival in patients with severe ischemic cardiomyopathy

**DOI:** 10.1186/1532-429X-13-S1-O91

**Published:** 2011-02-02

**Authors:** Deboarh H Kwon, Zoran B Popovic, Ernesto Ruiz Rodriguez, Carmel M Halley, Randall C Starling, Milind Y Desai, Thomas Marwick, Scott D Flamm

**Affiliations:** 1 Cleveland Clinic Foundation, Cleveland, OH, USA

## Introduction

Myocardial scar burden, measured by delayed hyperenhancement cardiac magnetic resonance (DHE-CMR), is an independent predictor of mortality in patients with ischemic cardiomyopathy (ICM) and severe left ventricular (LV) systolic dysfunction. It is unknown if myocardial scar burden impacts the protective benefit of implantable cardioverter defibrillators (ICDs).

## Purpose

In patients with severe ICM, we sought to assess the impact of LV myocardial scarring and ICD implantation on survival in patients with severe ICM.

## Methods

**Figure 1:**
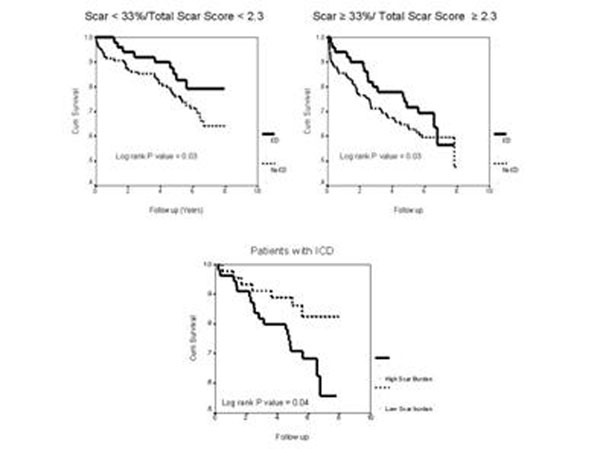


Patients (n = 337) with ≥ 70% disease in ≥1 epicardial coronary artery (77% men, median age 66 years, median LV ejection fraction [EF] of 22 %) undergoing CMR (Siemens 1.5-T scanner, Erlangen, Germany) between 2003-2007 were studied. CMR evaluation included long and short axis assessment of LV function on steady state free precession cine images along with assessment of myocardial scar (on inversion recovery DHE-CMR sequence ~ 10-20 minutes after injection of 0.2 mmol/kg of Gadolinium dimeglumine). Scar was identified as regions of interest > 2 SD above normal myocardium. LV scar was categorized based on mean scar % and transmural extent (0 = none, 1 = 1-25%, 2 = 26-50%, 3 = 51-75%, and 4 = > 75%). Total scar score was determined from the summed scar score of 17 segments per patient divided by 17. 94 patients underwent subsequent ICD implantation. The composite end-point was cardiac transplantation and all-cause mortality. Results: Over a follow-up of up to 8 years, there were 107 events (102 deaths, 5 cardiac transplantations). Receiver operating characteristic curve analysis was used to select the optimum threshold of mean scar % ≥ 33%, total scar score ≥ 2.3 to predict death/transplantation. On multivariate variate analysis, only age, total scar score, diabetes, gender, and ICD implantation were independent predictors of mortality/transplantation (Table 1). ICD resulted in significantly improved outcomes independent of scar burden (Figure 1 - log rank p = 0.03). However, amongst patients with ICD, mean scar % ≥ 33% or total scar score ≥ 2.3 was still a powerful predictor of mortality/transplantation (Figure 1 - log rank p = 0.04).

**Table 1:** Multivariate Analysis Demonstrating Independent Predictors of Survival in Patients with Severe ICM

	Hazard Ration (Confidence Interval)	P-value
**Age**	**1.04 (1.02-1.06)**	**0.00011**

**Total Scar Score**	**1.42 (1.16-1.73)**	**0.00048**

**Diabetes**	**1.91 (1.27-2.87)**	**0.00155**

**Gender**	**1.75 (1.13 - 2.71)**	**0.01**

**ICD**	**0.59 (0.36 - 0.96)**	**0.029**

HTN		0.50
Statin		0.89
Beta-blocker		0.36
ACE-inhibitor		0.98

Post-CMR CABG		0.48

Post-CMR MV repair/replecement		0.98

LV Ejection Fraction		0.26
LV End Diastolic Volume		0.24

## Conclusions

In patients with ICM and severe LV dysfunction, ICD provides mortality benefit, irrespective of myocardial scar burden. Nonetheless, scar burden remains a powerful predictor of mortality/cardiac transplantation even after ICD implantation.

